# The Use of Very High-Doses of Baclofen for the Treatment of Alcohol-Dependence: A Case Series

**DOI:** 10.3389/fpsyt.2014.00143

**Published:** 2014-10-10

**Authors:** Renaud de Beaurepaire

**Affiliations:** ^1^Groupe Hospitalier Paul-Guiraud, Villejuif, France

**Keywords:** alcoholism, addiction, treatment, side effect, abstinence

## Abstract

Baclofen, particularly high-dose baclofen, has recently emerged as a treatment of major interest for alcohol-dependence. However, baclofen has many potentially dangerous side effects, and the maximal dose of baclofen that may be used is a matter of discussion. Here, the author analyses the medical charts of the last 100 patients seen in his clinic, 17 of whom have been taking a very high dose of baclofen, which is to say, more than 300 mg/day. The analysis of the charts shows that the very high-doses baclofen were justified in almost all the cases. Side effects are analyzed.

## Introduction

High-dose baclofen has been shown to produce a remarkable state of indifference toward alcohol (1). However, the question of the upper limit of baclofen dosage that may be reached is a matter of discussion. Drug summaries recommend not exceeding the daily dose of 75–80 mg (75–80 mg/day) (2, 3) although long-term studies have shown that doses exceeding 250 or 300 mg/day are often necessary to produce a state of complete indifference to alcohol (4, 5). Recently, the French Health Safety Agency released a recommendation allowing for the prescription of baclofen to be given up to 300 mg/day, but not beyond, for the treatment of alcohol dependence (French Ministerial decree of June 13, 2014).

The present report is an analysis of the author’s medical charts of patients taking, or having taken, very high doses of baclofen, i.e., more than 300 mg/day for the treatment of their alcohol dependence. Since 2008, the author has prescribed baclofen to approximately 600 alcohol-dependent patients. The charts of the last 100 patients, followed for at least 6 months, were reviewed. At the time of the review (July 2014), all the patients except one (see Patient-14 below) were under the care of the author (last visit posterior to May 2014). Among the 100 charts, it appeared that 17 patients have been taking doses of baclofen superior to 300 mg/day at 1 time or another during their treatment (7 other patients reached the dose of 300 mg/day without exceeding it). This means that, according to this cohort, 17% of alcohol-dependent patients need more than 300 mg/day of baclofen for their treatment (and nearly one in four needs at least 300 mg/day).

The treatment was conducted similarly for all patients; doses were progressively increased (one additional 10 mg-tablet every 3 days) until a state of indifference toward alcohol (suppression of craving) was reached. After reaching a dose of 120–150 mg/day, if these doses were ineffective, or not sufficiently effective, patients were asked to actively participate in the dose increase. They were told that, unless they have no unbearable side effects, they must continue to increase the dose of baclofen, with no given limit of dosage, as long as they have episodes of craving for alcohol. In case of unbearable, or difficult to bear, side effects, they were told to slow the progression of the increase, or to decrease the dose until the side effect either disappears or becomes bearable, and then once again increase the dose, possibly much more slowly (one tablet, or a half-tablet, every week or 10 days, or possibly even more slowly). Patients were thereby trained to manage by themselves the increase of the doses according to a principle of self-involvement in the management of their treatment, while always under strict medical control (monthly visits, phone call to the author whenever necessary).

## Background

In previous reports of clinical cases of baclofen in alcohol-dependence patients were always treated with doses beneath 300 mg/day (1, 6–8).

## Clinical cases

Clinical cases are presented below (see also Table 1). Patients are classified according the length of their follow-up, from the longest to the shortest. Side effects will be considered in a separate paragraph in the Section “Discussion.”

**Table 1:** **Digest of cases**.

Patient	Year of birth	Gender	Pretreatment daily alcohol consumption, in grams	Number of years of alcohol dependence before treatment	Baclofen starting date (and duration of treatment, in months)	Maximal baclofen dose, in mg	Duration of dose over 300 mg/day, in months	Last visit baclofen dose, in mg	Last visit alcohol consumption, in mg (and stability)
Pt1	1950	M	240	20	17/12/2008 (67)	360	1	360	0 (Stable)
Pt2	1981	M	450	6	17/06/2009 (60)	320	3	300	20–30 (Stable)
Pt3	1951	F	240	6	25/03/2010 (51)	400	3	300	<10 (Stable)
Pt4	1978	M	350	10	18/05/2010 (49)	520	11	320 (Irregular)	0 (Most of time)
Pt5	1978	M	240	10	23/06/2010 (48)	400	2	250 (Irregular)	120 (Irregular)
Pt6	1972	F	310	3	03/11/2010 (44)	330	9	330	0 (Stable)
Pt7	1981	M	380	12	17/08/2011 (39)	320	8	300	150 (Weekend only)
Pt8	1967	M	280	22	19/08/2011 (39)	420	5	400	20 (Stable)
Pt9	1969	M	320	15	11/01/2012 (26)	440	22	390	20 (Stable)
Pt10	1955	M	180	10	05/12/2012 (19)	360	1.5	360	50 (Irregular)
Pt11	1961	M	280	20	09/01/2013 (18)	420	13	300	60 (Stable)
Pt12	1963	M	350	15	12/04/2013 (14)	320	1	320	120 (Most of time)
Pt13	1976	M	180	8	03/09/2013 (10)	350	1.5	200	0 (Stable)
Pt14	1975	M	230	3	12/11/2013 (6)	630	1.5	630	0 (But relapsed)
Pt15	1984	M	350	5	02/12/2013 (7)	400	2.5	320 (Irregular)	0 (Most of time)
Pt16	1972	M	200	10	18/12/2013 (7)	310	0.5	290	30 (Irregular)
Pt17	1982	M	240	6	20/12/2013 (7)	310	2	310	0 (Stable)

### Patient-1

Patient-1 (Pt1) is a 58-year-old healthy farmer drinking approximately three or four bottles of wine per day (he denied drinking, hiding bottles in the bush). He was brought in our clinic by his family, and did not seem to be very motivated to stop drinking. He was forced to take the baclofen treatment, given authoritatively by his wife, and rapidly drank much less (at 120 mg/day), but nevertheless continued to drink to excess (on the sly), as evidenced by biological measures. He stopped drinking at 210 mg/day (normalization of hepatic enzymes). After a year or two, he decided he was cured, stopped baclofen, and relapsed a few months later. Baclofen was restarted but the dose of 210 mg was insufficient. Doses were then increased (in large part driven by his daughter who was a pharmacist) and he was taking 360 mg/day at the time of his last visit (June 2014). According to the family, he has no craving at all, not drinking even when he is offered a glass of wine.

### Patient-2

Patient-2 (Pt2) is a 28-year-old salesman drinking one to two bottles of whiskey per day (4–8 20 cc whiskey flasks), and a variable number of beers. He is emotionally very unstable, impulsive, abuses cannabis, and has a long history of anxiety and depression for which he has previously been treated but he was taking no psychotropic medications (except benzodiazepines) when baclofen was started. The road to abstinence with baclofen turned out to be very long and chaotic, with nearly 4 years of ups and downs. Baclofen was initially very effective; he stopped drinking completely at 120 mg/day but started drinking again after a month or two, beer only. He later turned to sparkling wine for several months. He alternated periods of sobriety, periods of moderate drinking (one bottle of sparkling wine or some beer) and short episodes of massive drinking during the following years. He acknowledged that baclofen greatly reduces his craving, and he has progressively increased it over the years, reaching the dose of 320 mg during some months in 2013. But his daily treatment compliance was irregular (often off-schedule). He is now (June 2014) taking 300 mg/day regularly, he says, and drinks an average of two or three beers per day. He is still impulsive and equally emotionally unstable and is still cannabis-dependent (although his consumption of cannabis has substantially decreased).

### Patient-3

Patient-3 (Pt3) is 59-year-old woman referred for an intractable alcoholism (4 bottles of white wine per day) associated with a fronto-temporal dementia. The dementia was labeled idiopathic by her neurologist, independent of the alcoholism. She is treated by clomipramine and donepezil. Baclofen was considered by the family as a last-resort attempt. Increasing baclofen doses proved to be extremely difficult due to the massive memory problems of the patient and to insufficient surveillance of treatment administration by the (very busy) family. It took 2 years to reach the dose of 400 mg/day (thanks to an ancillary worker who was hired by the family to accompany the patient and control her treatment observance). At that dose, the patient suddenly stopped drinking, showing a total indifference toward alcohol. Two years later, she is still sober (ritually filling at dinner time a glass of wine, which she does not finish), and has recovered fairly good memory capacities (the diagnosis of idiopathic dementia has been abandoned).

### Patient-4

Patient-4 (Pt4) is 32-year-old technician drinking one bottle of rum every day. He suffers from a severe anxiety disorder, with agoraphobia and emotional instability (treated with an antidepressant and an atypical antipsychotic). He became almost completely abstinent at 200 mg/day (although not completely indifferent to alcohol), but had episodes of relapse due to stress and instability (mainly in relation to conflicts in the workplace, a divorce and concomitant painful somatic illness). He shifted from rum to beer, and increased baclofen doses at each relapse, reaching the dose of 520 mg/day. He is presently stabilized at 320 mg/day, does not drink and says he has no craving, although still having brief episodes of massive alcoholization (an average of one every 2 months), when he drinks an entire bottle of rum and/or other drinks over 1 or 2 days.

### Patient-5

Patient-5 (Pt5) is 32-year-old unemployed artist-painter drinking an average of three bottles of wine and 1 l of beer per day. He has a long history of untreated anxiety and depression. He takes no medications except occasional benzodiazepines. Alcohol has an obvious anxiolytic and antidepressant function for him, and his real desire to stop drinking was questionable when he started baclofen. His craving for alcohol and alcohol consumption significantly decreased (one bottle of wine, no more beer) at a relatively low dose of baclofen (150 mg/day). However, partly due to stressful life events, he progressively relapsed and slowly resumed his previous drinking habits, taking less and less baclofen until a complete stop that lasted several months. He then restarted baclofen, acknowledging that although baclofen greatly decreases his craving, it is, however, ineffective in completely suppressing his compulsive rituals in drinking. He slowly and irregularly increased baclofen up to 400 mg/day, remaining at that dose for approximately 2 months, still drinking to excess (one to two bottles of wine per day), after what he decreased the dose of baclofen down to 250 mg/day, which he is still taking, not very regularly, still drinking an average of 1.5 bottle per day.

### Patient-6

Patient-6 (Pt6) is a 38-year-old Human Resources assistant drinking half a bottle of gin and a bottle of wine every day. She is healthy and takes no medications. She stopped drinking at the dose of 240 mg/day of baclofen. But 2–3 months later, she started to drink moderate doses of alcohol again and increased the baclofen dose to 270 mg/day. She remained 2.5 years taking this dose very regularly and was almost always sober, but she had more and more often moments of craving when coming home from work in the evening and got into a habit of adding three more tablets to the evening dose, and after a while she added another three tablets. She is now taking baclofen 330 mg/day, very regularly. She is sober and has no craving.

### Patient-7

Patient-7 (Pt7) is a 30-year-old commercial employee drinking one or more bottle of rum every day. He suffers from panic attacks (no treatment, except benzodiazepines). He immediately appreciated baclofen, finding that it gave him a feeling of euphoria with a marked decrease in anxiety. He rapidly stopped drinking during the week but continued to drink with friends during the weekend. After a few months, while he was regularly taking 200 mg/day, he started drinking again, as before, during the week. He then progressively increased baclofen up to 400 mg/day and stopped drinking during the week though continuing to drink during the weekend (more as a routine than because of a craving). He is now stabilized at 300 mg/day, does not drink during week days (no craving), but still drinks during the weekend (a bottle of rum or whiskey for the 2 days), and often decreases the dose of baclofen during the weekend “to facilitate drinking” he says. (The decrease of baclofen is often accompanied by a baclofen withdrawal syndrome, marked by sweats, anxiety, shaking, and itching – an unusual syndrome during baclofen partial withdrawal).

### Patient-8

Patient-8 (Pt8) is a 44-year-old city hall employee, drinking an average of 5 l of beer every day and a variable quantity of wine. He has a long history of depression and suicide attempts, detox cures and psychotropic treatments. At the beginning of baclofen treatment, he was taking an antidepressant and several anxiolytics. Baclofen dose increase was slowed by the occurrence of many difficult to bear side effects. He started decreasing alcohol consumption at 200 mg/day. At 250 mg/day, he had almost no more craving, but continued compulsively to drink during what he called his “evening rituals”: one beer and two to four glasses of wine. This “ritual” consumption lasted 18 months, during which the patient was regularly taking 250 mg/day of baclofen after which, following certain life events, he decided to stop drinking completely, and on his own initiative increased baclofen to this end. He reached the dose of 420 mg/day, at which he stopped drinking completely. He then decreased the dose to 400 mg/day and is still at that dose in July 2014, still sober.

### Patient-9

Patient-9 (Pt9) is a 43-year-old invalid man, drinking one bottle of whiskey per day, always in the evening. He has a history of bipolar disorder (treated by depakine + olanzapine + escitalopram + several benzodiazepines), and is a former cocaine and cannabis addict. Baclofen was progressively increased up to 300 mg/day without any effect. Above this dose, Pt9 began to progressively decrease drinking and to change his drinking habits, shifting from whiskey to rosé wine, which he began at noon. At 350 mg/day he drank an average of 1.5 bottle of wine, at 440 mg/day a half bottle, and at 480 mg/day (eleventh month of treatment), only two glasses. Seven months later, he was still taking 480 mg/day, was sober, but said he still has frequent episodes of craving. He nevertheless started to decrease the dose of baclofen and, at 200 mg/day, had a long period of relapse for which he needed to be hospitalized. After that, he again increased baclofen progressively, up to 400 mg/day. He now takes 390 mg/day (June 2014), and drinks an average of two glasses of wine per day. He feels very well, and plans to take up volunteer work.

### Patient-10

Patient-10 (Pt10) is a 57-year-old pharmacy technician drinking three bottles of wine per day. He is in good health and takes no medications. The dose of baclofen was progressively increased up to 320 mg/day over 7 months, without incident, and the consumption of alcohol decreased by more than two thirds (daily amount between ½ and 1 bottle per day), with a craving notably reduced but still present. The length of treatment, 7 months, is probably insufficient, and it is expected that a further dose increase will allow the achievement of a complete suppression of craving.

### Patient-11

Patient-11 (Pt11) is a 52-year-old bricklayer (on sick leave for the last 2 years) drinking 4–5 l of beer (7.5°) per day. He is also a cannabis addict and suffers from depression (taking paroxetine over the last 5 years). His craving was almost completely suppressed at 200 mg/day but there remained strong rituals of drinking that made him continue to drink despite an obvious lack of interest for alcohol. He spontaneously increased progressively the doses up to 420 mg, and stopped drinking completely at that dose, but his personal attending physician told him that the dose was too high and potentially dangerous (despite an almost complete absence of side effects) and so he decreased the dose. He is now taking 300 mg/day and drinks an average of 1 l of beer per day. He returned to work and takes no more paroxetine.

### Patient-12

Patient-12 (Pt12) is a 50-year-old insurance employee drinking a bottle of pastis (a 45° beverage close relative of absinth) every day. He is in good health and takes no medications. Baclofen increase proved to be very difficult because of the occurrence of a number of unbearable side effects at relatively low doses of treatment. He nevertheless very courageously surmounted these effects and slowly increased the doses. The nature of side effects changed at higher doses (from delirium and serious behavioral disturbances to slowness, bizarre somatic sensations, sweating, and insomnia). The first sign of a decrease in drinking (280 mg/day) was that he began to drink more and more slowly, keeping the beverage several minutes in the mouth before swallowing (this is a rare, but not exceptional, effect of baclofen in alcoholics). He progressively decreased alcohol consumption but did not stop it, being strongly and compulsively attached to his evening drinking habits. The alcohol consumption nevertheless decreased with the increase of baclofen. At the last visit, the dose was 320 mg/day. He was still drinking approximately the third of a bottle of pastis every day, and he planned to continue to increase baclofen. Bromazepam was added to his treatment in August 2013, for insomnia, which he is still taking (and which is a great help).

### Patient-13

Patient-13 (Pt13) is a 37-year-old supermarket manager drinking half a bottle of whiskey per day. He is in good health. Baclofen progressive increase was very slow due to the occurrence of many uncomfortable side effects. Baclofen was increased up to 300 mg/day without any effect on alcohol consumption or craving. Suppression of craving and alcohol consumption occurred abruptly at 350 mg/day. Pt13 takes all baclofen tablets at once in the morning before going to work (a very unusual way of proceeding, but Pt13 found this was the best for him). He took this dose for a month or two, then decreased it to 300 mg, then to 200 mg/day. In July 2014, he had no craving, was totally abstinent but progressively developed a worrying depression over the last months. Whereas he was taking no medication when baclofen was started, he is now taking escitalopram, alprazolam, and zopiclone.

### Patient-14

Patient-14 (Pt14) is a 38-year-old salesman (unemployed) drinking an average of 5 l of beer per day. He drinks a little during the day but drinks massively at evening and night-time, away from home, with friends, compulsively until totally drunk. His wife is threatening him with divorce. He is very impulsive, suffers from a major anxiety disorder, and takes no medication. He increased progressively baclofen, and began to decrease drinking at 250 mg/day. At 360 mg/day (fourth month of treatment), he was not drinking more than two or three cans of beer. He suddenly decided (without medical advice) to increase baclofen rapidly until he reaches total abstinence. Total abstinence occurred at the dose of 630 mg/day. He remained at this dose for 1.5 months, despite medical injunctions to decrease the dose. He found a job and seemed extremely satisfied: “my wife is happy,” “my life has completely changed.” At the end of May 2014, he showed signs of an acute delirium, with agitation in the street and a facial hematoma, and was transported to an intensive care unit by a police emergency squad. He regained consciousness a few hours later, and left the unit against medical advice <24 h after admission. He has given no news since despite many attempts to reach him. He very likely stopped baclofen.

### Patient-15

Patient-15 (Pt15) is a 29-year-old successful business manager drinking one bottle of whiskey per day, and often some other drinks. He is healthy and takes no medications. Detox cures and usual medications were ineffective. He escalated doses more rapidly than planned, became indifferent to alcohol at 250 mg/day, but deliberately increased the dose up to 400 mg/day (“to be on the safe side” he said), and then decreased the dose, maintaining it most often around 320 mg, sometimes higher (in case of stress), sometimes much lower (when he decides to “live it up” with friends). He is almost always sober and has no craving. This case is a typical case of successful self-management of the treatment.

### Patient-16

Patient-16 (Pt16) is a 41-year-old salesman in a fashion boutique, drinking mostly beer (an average of 3 l/day), but also any kind of alcohol when accessible. He is also cannabis dependent, and occasionally takes cocaine. He is impulsive, very unstable emotionally, and often has severe fits of anger and aggressiveness. He appeared to be unable to increase progressively and regularly the doses of baclofen as prescribed. He increased baclofen very rapidly to 270 mg/day (“as Olivier Ameisen did” he said), then decreased it rapidly, increased once again the doses, reaching 310 mg/day for a short while. He says that baclofen has a stimulant effect on him, and makes him feel more placid and less aggressive. He sometimes seems to take baclofen as if it were a drug of abuse, but he also says that alprazolam or hypnotics make him “feel high.” After 6 months of more or less chaotic, but constant, baclofen treatment, he appears to have almost no craving for alcohol, drinks rarely (only when driven by friends), has substantially decreased cannabis, and has started a treatment for his tobacco dependence.

### Patient-17

Patient-17 (Pt17) is a 31-year-old engineer drinking an average of 60 cc of whiskey or vodka per day. He suffers from depression (treated with paroxetine). Drinking progressively decreased with the increase of baclofen. He reached a complete suppression of craving at 310 mg/day. He had a brief relapse 2 months ago (sudden compulsive drinking of a flask of vodka, for no particular reason), and is totally sober since.

## Discussion

This retrospective analysis of medical charts shows that a substantial number of alcohol-dependent patients treated with baclofen need treatment doses superior to 300 mg/day (17% of the 100 cases). It is clear that if the doses of baclofen had been limited to 300 mg/day as recently recommended by the French Health Safety Agency, these patients would not have fully benefited from the craving–suppressing effects of baclofen.

The clinical vignettes show that the use of high-dose baclofen took place in many different contexts and situations, sometimes potentially inappropriate [family pressure (Pt1), or patient’s impulsivity (Pt14)], but in most cases the high-dose treatment was justified, resulting in very positive effects.

Side-effects occurred in all the patients except Pt4, who always denied any side effect (which one may doubt). Side effects were in general minor and benign. Almost all patients reported fatigue and/or insomnia. Insomnia was sometimes severe. Most of the patients had insomnia before baclofen, but baclofen itself induced or worsened insomnia in many of them (baclofen-induced insomnia was often improved by hypnotics but not always, however). Other benign side effects were nausea, dizziness, headache, pain in muscles and joints, motor instability, feeling of head crushing, feelings of electric discharges, decrease in vision, decrease in libido, aggressiveness, transient hypomania, paresthesias, tinnitus, sweats, cramps, spasms, dry throat. There were four cases of severe side effects: Pt8 was suffering from somnambulism (and falls during sleepwalking) long before treatment, but baclofen worsened his somnambulism, which became associated with nocturnal delirium, and 1 day Pt8 fell during an episode of sleepwalking and broke his ankle (at that time, baclofen dose was 370 mg/day, and he was drinking about 50 g of alcohol per day). Pt12 had a period of nocturnal delirium associated with bizarre stereotyped behavior during the day, and 1 day he woke up with paralyzed legs. The emergency physician suspected a stroke and he was hospitalized, but stood up normally a few hours later. All the investigations (cardiovascular, brain imaging) were normal (at that time, baclofen dose was 210 mg/day, and he was drinking more than 300 g of alcohol per day). Pt13 progressively developed a depressive state, which, although Pt13 has no suicidal thoughts, could be considered as a serious side effect (the direct relationship with baclofen is, however, disputable). Pt14, as described in the clinical vignette, took an unreasonable amount of baclofen, and 1 day presented a state of confusion and a facial hematoma and was hospitalized in an intensive care unit for that, but the precise circumstances of this severe side effect have not been elucidated.

## Concluding Remarks

Baclofen treatment is for many alcoholic patients a long story and a long fight. The fight involves both the patient and the physician, and the notion of therapeutic alliance finds here all its meaning, as it is essential that the patient participates in the managing of the treatment under the control of the physician. To be effective, baclofen may need to be given at high or very high doses. An imposed limit to the dose of baclofen is a loss of the opportunity of being cured for many patients. Baclofen is not more dangerous at high doses than at low doses when the treatment is well supervised by the physician. Side effects, even severe side effects, can occur at any dose. Most of the time, difficult to bear side effects present at low doses will vanish or change in nature when the baclofen dose is increased. Therefore, on the condition of a good therapeutic alliance between the patient and the physician, baclofen should be prescribed with no imposed upper limit of dosage.

## Conflict of Interest Statement

The author declares that the research was conducted in the absence of any commercial or financial relationships that could be construed as a potential conflict of interest.
